# Editorial: Advances in neurocritical care

**DOI:** 10.3389/fmed.2026.1971457

**Published:** 2026-09-02

**Authors:** Linlin Zhang, Jian-Xin Zhou

**Affiliations:** 1 Department of Neurocritical Care, Beijing Anzhen Hospital, Capital Medical University, Beijing, China; 2 Department of Critical Care Medicine, Beijing Shijitan Hospital, Capital Medical University, Beijing, China

**Keywords:** acute brain injury, analgesia and sedation, delirium, extubation, fluid therapy, mechanical ventilation, neurocritical care, quality control

## Introduction

Neurocritical care focuses on patients with actual or impending organ dysfunction that arises from, or accompanies, primary or secondary neurological injury and necessitates intensive care (1). As a subspecialty, neurocritical care continues to face unresolved and controversial questions in both clinical practice and research, many of which require further investigation. Chen provide a broad overview of recent developments in severe nervous systematic diseases, neuromonitoring, hemodynamic and respiratory support, and post-cardiac-arrest car (1). Within this broader landscape, the present Research Topic, Advances in Neurocritical Care, examines more focused clinical, physiological, diagnostic, and methodological questions across these domains. It comprises 29 contributions: 15 research articles (14 Original Research articles and one Clinical Trial), three Systematic Reviews, three Reviews, two Study Protocols, and six Case Reports. The contributions are discussed here under four overlapping themes: dynamic physiological monitoring; biomarkers, measurement, and prediction; perioperative and neurocritical care management; and systemic, metabolic, or treatment-related neurological injury.

### Why trajectories may matter

Continuous monitoring derives clinical value from its integration with bedside assessment. Serial clinical examination remains indispensable, with trajectories serving as one component of repeated neurological assessment and contextual interpretation. Automated pupillometry has gained wider use because it provides an objective, repeatable measure of pupillary reactivity. Chen highlighted the ORANGE cohort, in which abnormal Neurological Pupil Index (NPi) measurements were associated with unfavorable neurological outcomes and higher mortality after severe non-anoxic acute brain injur (1). The 2025 B-ICONIC Brussels consensus proposed an NPi of 3 or lower as suggestive of intracranial hypertension in traumatic brain injury (TBI) when invasive intracranial pressure (ICP) monitoring is unavailable and recommended integrating NPi with the clinical examination and at least one additional non-invasive modality (2). Kim et al. followed serial NPi values during the first 72 h after non-traumatic subarachnoid hemorrhage and found lower early NPi values in patients with unfavorable outcomes. These findings support serial assessment, although an intervention threshold remains to be defined. A low or falling NPi warrants repeat examination and review of imaging, physiology, and potential confounders before a protocolized response is initiated.

Cerebral perfusion pressure (CPP) trajectories raise a related question. Wang et al. identified four phenotypes among 1,466 patients and found the highest mortality in the rapidly declining group. The gain in discrimination was modest. Earlier CENTER-TBI work linked time below an individualized lower limit of reactivity with mortality, while COGiTATE showed that autoregulation-guided CPP targeting was feasible and safe (3, 4). Wang et al.'s study broadens the population beyond TBI. Its contribution lies in identifying how often and in whom CPP deteriorates. The appropriate bedside response to that pattern remains to be established prospectively.

Positioning after craniotomy and hypotension during non-cardiac surgery may appear to be separate issues, yet both studies show why a threshold stripped of time and context is incomplete. In 21 postoperative patients, Li et al. found that a flat head-of-bed position increased ICP and reduced CPP, while tissue oxygenation remained stable. Baseline pressure and autoregulatory status altered the response. The brief physiological observation leaves the effect of position on recovery unresolved and cautions against assuming a uniform response. Ren et al. analyzed minute-by-minute arterial pressure in 789 operations. Complications increased as hypotension became sustained, prolonged, or fluctuating. Here, the clinically relevant exposure was the temporal pattern of hypotension.

Signal quality is the central problem in two other monitoring studies. Hinsberger et al. traced most difficulties in electroencephalographic assessment during brain death determination to technical artifacts, especially electrode-related artifacts. The contribution of electroencephalography depends on compliance with technical standards and the applicable jurisdictional protocol. Jiang et al. evaluated diaphragm ultrasound in 188 neurosurgical intensive care unit (ICU) patients. First spontaneous breathing trial (SBT) success and first extubation success were similar across study phases, although ultrasound-guided phases had fewer reintubations and shorter ventilation. The sequential design makes the size of that benefit uncertain. The larger issue, from our perspective, is conceptual: diaphragm thickening fraction captures respiratory muscle performance, whereas airway protection depends on a broader set of functions. Current consensus treats extubation after acute brain injury as more than a respiratory test (5). Xu et al. had already operationalized that distinction in 226 neurosurgical patients: their STAGE score combined swallowing, tongue protrusion, spontaneous and suctioning cough, and the Glasgow Coma Scale motor response, and showed moderate discrimination for extubation success, with an area under the curve (AUC) of 0.72 (6). Badenes et al. later framed the same shift from respiratory load to airway protection, noting that, once an SBT is passed, vigorous cough may matter more than the SBT modality (7). We would therefore integrate diaphragm ultrasound with assessment of cough, secretion burden, consciousness, and bulbar function. Passing an SBT demonstrates short-term unsupported breathing capacity; airway safety requires separate evaluation.

The Chinese neonatal extracorporeal membrane oxygenation (ECMO) consensus has a different purpose. Lu et al. describe how a Chinese expert consensus on neurological monitoring and long-term follow-up will be developed using a systematic review and the Grading of Recommendations Assessment, Development and Evaluation (GRADE) approach. We welcome its inclusion of neurodevelopment after discharge; survival alone is an incomplete outcome in this population. The protocol describes the methods, while the clinical recommendations and their feasibility await completion of the consensus process.

### Biomarkers and prediction: the gap between association and use

Wang et al. studied the cerebrospinal fluid glucose-to-lactate ratio in 121 postoperative patients with acute brain injury and suspected intracranial infection. The area under the receiver operating characteristic curve was 0.866, with similar performance across glycemic strata. Because both measurements are routinely available, the ratio has practical appeal. We are less interested in the reported decimal than in whether the cut point survives changes in prior antibiotics, sampling time, case mix, and the reference diagnosis. Those details decide whether the test can travel.

Three systemic biomarker studies remain further from a treatment decision. In 5,267 critically ill patients with stroke from the Medical Information Mart for Intensive Care IV (MIMIC-IV), Wang et al. found a graded relationship between the leuko-glycemic index and mortality and reproduced it in an institutional cohort of 424 patients. Another team serially measured soluble triggering receptor expressed on myeloid cells 1 and 2 (sTREM-1 and sTREM-2) in 120 patients after cardiac arrest and incorporated the measurements into machine-learning models. Kashatnikova et al. examined T-cell receptor excision circles and B-cell K-deleting recombination excision circles during rehabilitation after TBI; the recruitment setting leaves the acute phase and patients who never reached rehabilitation outside the frame. We read all three as candidate biological phenotypes.

Xia et al. and Wang et al. developed clinical prediction tools that utilize common variables to assess postoperative delirium after traumatic cervical spine surgery and pulmonary infections after cerebral hemorrhage. Their simplicity is attractive, but delirium screening, extubation practice, antibiotic use, and definitions of pneumonia differ between hospitals. Zhang et al.'s Consensus-based Standards for the Selection of Health Measurement Instruments (COSMIN) review of the revised Richards-Campbell Sleep Questionnaire points to an even earlier problem: several language versions lack adequate evidence for measurement properties beyond internal consistency. A cleaner algorithm cannot rescue an unstable label. Before adding predictors, investigators need to show that the same outcome is being measured at the same time in the same way.

### Translating evidence into perioperative and neurocritical care management

Fluid choice in neurocritical care requires condition-specific interpretation of evidence derived from general ICU populations. In a secondary analysis of the BaSICS randomized trial, patients with TBI assigned to Plasma-Lyte 148 had a high probability of greater 90-day mortality than those assigned to saline; the subgroup design limits any class-wide inference about balanced solutions (8). The 2018 European Society of Intensive Care Medicine (ESICM) consensus recommended isotonic crystalloids for maintenance and resuscitation in acute brain injury and advised against hypotonic solutions; albumin was not recommended in TBI (9). The 2024 ESICM clinical practice guideline similarly emphasized tonicity and condition-specific selection, reflecting clinically important differences among balanced crystalloids (10). In this context, Duan et al.'s analysis of balanced crystalloid use in subarachnoid hemorrhage is clinically relevant. Its observational design leaves the optimal formulation, dose, and patient phenotype unresolved. For now, fluid should be prescribed as a drug: by indication, composition, dose, cumulative balance, and neurological context.

Analgesia and sedation are particularly contentious after neurosurgery. Sedatives can obscure serial consciousness assessment and may delay recognition of hemorrhage, ischemia, or impending herniation; insufficient treatment of pain and agitation can also worsen physiological stress and expose patients to device removal or secondary injury. The Chinese expert consensus therefore recommends individualized targets according to brain injury, intracranial dynamics, ventilation, procedures, and the need for neurological assessment (11). Wang et al. describe a single-center, single-arm feasibility protocol without a randomized comparator. In 65 selected adults after craniotomy who are restless or agitated but do not require deep sedation, non-pharmacological measures and remifentanil-based analgesia are titrated to Richmond Agitation-Sedation Scale (RASS) scores of −2 to +1 and Critical-Care Pain Observation Tool (CPOT) scores of 0–1; midazolam or propofol remains available as rescue therapy. The primary endpoint is successful protocol management during the first 24 h. The protocol is designed to assess feasibility and safety. Its full report should clarify the frequency of rescue sedation and whether neurological assessment remains feasible without avoidable agitation or physiological harm.

The two delirium studies warrant cautious interpretation. Sun et al. reported less postoperative delirium with preoperative warming plus dexmedetomidine in 153 analyzed older adults undergoing hip-fracture surgery, but some randomized participants were excluded and the setting was not neurocritical care. Dong et al. tested a virtual-reality package after cardiac surgery; only eight delirium events occurred among 40 participants. The package appeared feasible to deliver. However, orientation, sleep support, mobilization, and the additional staff attention may account for part of the observed effect.

Temperature management illustrates the distinction between evidence synthesis and comparative treatment evidence. Using a 6S evidence framework, Zhang et al. appraised 20 sources (seven guidelines, six expert consensuses, four systematic reviews, two evidence summaries, and one clinical decision resource) and summarized 27 best-practice items across preparation, initiation, maintenance, complication management, rewarming, and prognostic management. Its design supports an implementation-oriented synthesis across heterogeneous neurological conditions; comparative treatment effects lie outside its scope. For comatose adults after out-of-hospital cardiac arrest, the Targeted Hypothermia Versus Targeted Normothermia After Out-of-Hospital Cardiac Arrest (TTM2) trial found no mortality or functional benefit from induced hypothermia at 33 °C compared with targeted normothermia and early fever treatment (12). European Resuscitation Council–European Society of Intensive Care Medicine (ERC–ESICM) guidance emphasizes continuous core-temperature monitoring and active fever prevention for at least 72 h, while finding insufficient evidence for or against a 32–36 °C target (13). Kortli and Nasa similarly emphasize post-resuscitation care as a bundle rather than a temperature target alone. The practical focus is the quality of temperature control: safe delivery, fever prevention, and delayed multimodal neurological prognostication (14, 15).

The review of decompressive hemicraniectomy pools 14 randomized trials and 1,003 patients with malignant middle cerebral artery infarction. Survival improved, while the functional interpretation varied with age, follow-up, and the chosen threshold; some analyses counted modified Rankin Scale scores of 0–4 as favorable. That definition matters to families. The decision and timing of decompression remain diagnosis-specific. Chen highlighted the Randomized Evaluation of Surgery with Craniectomy for Patients Undergoing Evacuation of Acute Subdural Haematoma (RESCUE-ASDH) trial, in which patients undergoing evacuation of a traumatic acute subdural hematoma were randomized intraoperatively to replacement or non-replacement of the bone flap. Disability and quality of life at 12 months were similar; decompressive craniectomy reduced early additional cranial operations but increased wound complications (1, 16). These findings apply specifically to the bone-flap decision during acute subdural hematoma evacuation; prophylactic decompression follows a different evidence base. For malignant middle cerebral artery infarction, European Stroke Organisation (ESO) guidance supports surgery within 48 h in adults aged 60 years or younger, with greater uncertainty in older patients and after 48 h (17). Hu et al. update these estimates within the populations represented by the available evidence. Karam et al. writing about blood stewardship and quantitative futility assessment in bleeding neurotrauma, expose the ethical counterpart of the same problem. When used to guide treatment limitation, prediction tools can create self-fulfilling bias. The appropriate safeguard is repeated clinical assessment combined with multidisciplinary discussion and an explicit account of the patient's values.

### When neurological deterioration originates outside the brain

Hyponatremia after neurological injury requires a mechanistic diagnosis that extends beyond the laboratory value. A meta-analysis by Li et al. of 12 observational studies involving 2,355 patients with acute spinal cord injury found associations with upper cervical or complete injury, hypoproteinemia, and infection. Two TBI case reports show why those associations alone are insufficient to guide treatment. Li et al. describe cerebral salt wasting syndrome with hypovolemia; Liu et al. describe hypopituitarism with secondary adrenal insufficiency. Fluid restriction, volume repletion, sodium replacement, and hormone replacement lead in different directions. For quality control, a sodium-correction metric without documentation of volume status, serum and urine indices, drug exposure, and pituitary-adrenal function would reward the number while missing the diagnosis.

The remaining case reports are best read as diagnostic interruptions. Zhou et al. describe late-onset ornithine transcarbamylase deficiency with cerebral edema and delayed worsening despite early ammonia normalization; Liu et al. report valproate-induced hyperammonemic encephalopathy after aneurysm clipping. Lu et al. observed acute pancreatitis during therapeutic hypothermia in two patients, and Chen report a deformed peripherally inserted central catheter with upper-extremity thrombosis after post-craniotomy seizures. The details are memorable because they interrupt a familiar explanation of neurological decline. Their clinical role is targeted investigation when the exposure and clinical pattern fit; routine population-wide screening is unsupported.

### What is ready for practice?

Only a limited set of messages can be transferred directly into current care, most of them already consistent with established guidance. At the bedside, quality control begins with technically valid monitoring and documented assessment of pain, sedation, and delirium. Extubation assessment combines ventilatory capacity with airway-protection variables, while fluid and electrolyte therapy requires explicit indications and targets. After cardiac arrest, implementation centers on fever prevention, delayed prognostication, management of major confounders, and integrated interpretation across modalities. These auditable processes are the principal practice-ready implications of the Research Topic.

Other contributions remain at different stages of investigation. Studies of pupil and CPP trajectories, autoregulation-guided positioning, and diaphragm-ultrasound-directed extubation should first define the bedside action linked to each signal and then evaluate that action in a controlled design. For diagnostic cut points and prediction models, the immediate priority is external testing with harmonized time zero, outcome definitions, and calibration. Patient-centered comparative trials are the next step for balanced fluids, analgesia-first sedation, warming plus dexmedetomidine, and virtual-reality interventions. Artificial intelligence (AI)-assisted clinical decision support requires a distinct pathway centered on prospective technical validation, workflow integration, and safety monitoring. Those are different research jobs; calling all of them “promising” hides the distinction.

### Priorities for a second edition

A recurring question while reading this collection was simple: what would the clinician do differently when a monitor changes? Too few observational studies specify the action that a signal is meant to trigger. Future work should place measurement, treatment, and outcome on the same time axis, particularly for pupillometry, pressure, oxygenation, electroencephalography, respiratory variables, and treatment exposures. Clinical value emerges when a dynamic phenotype identifies a modifiable state or differential treatment response; relabeling severity is insufficient.

The modeling papers raise a practical question: when does the clinical clock start? Each protocol should prespecify time zero and the prediction horizon for a specific decision, then evaluate the effects of data latency, artifacts, and missingness within that workflow. Prospective assessment of an AI system could begin with silent deployment of a locked model to verify data integrity, timestamp alignment, interpretability, and alert burden. A subsequent small human-in-the-loop pilot could assess clinician overrides, subgroup-specific failures, and performance drift (18, 19). Prognostic models require additional safeguards against self-fulfilling bias introduced by treatment-limitation decisions.

Our priorities for a second edition of *Advances in Neurocritical Care* focus on multicenter evaluations of monitoring-triggered interventions and bedside pathways that capture both benefit and harm. Eligibility and the trigger-linked action should be prespecified, together with reassessment intervals, protocol deviations, and stopping criteria. Designing these initiatives as both research and quality-improvement programs would provide a more credible route from physiological signals to better neurocritical care.
